# Supplementary material for: The C-terminal tail of α-synuclein protects against aggregate replication but is critical for oligomerization
Source: Commun Biol. 2022 Feb 10;5:123. doi: 10.1038/s42003-022-03059-8 (PMC8831632; doi:10.1038/s42003-022-03059-8)
Supplement: Supplementary file 2 — Supplementary Information [file 42003_2022_3059_MOESM2_ESM.pdf]

The C-terminal tail of α-synuclein protects against aggregate replication but is critical for oligomerization

Azad Farzadfard, Jannik Nedergaard Pedersen, Georg Meisl, Arun Kumar Somavarapu, Parvez Alam, Louise Goksøyr, Morten Agertoug Nielsen, Adam Frederik Sander, Tuomas P.J. Knowles, Jan Skov Pedersen and Daniel Erik Otzen

Supplementary Information

SI FIGURES

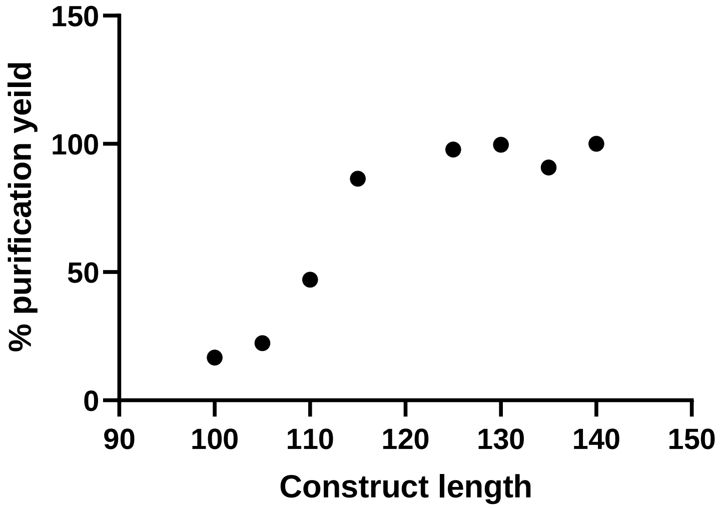

Figure S1. Expression yield of purified αSN decreases steeply for αSN110 and shorter mutants. Anion exchange chromatography was used for constructs αSN-FL to αSN121, and cation exchange chromatography was used for shorter constructs due to the decline in net negative charge. αSN-FL typically yields 30-40 mg purified αSN per L bacterial cell culture.

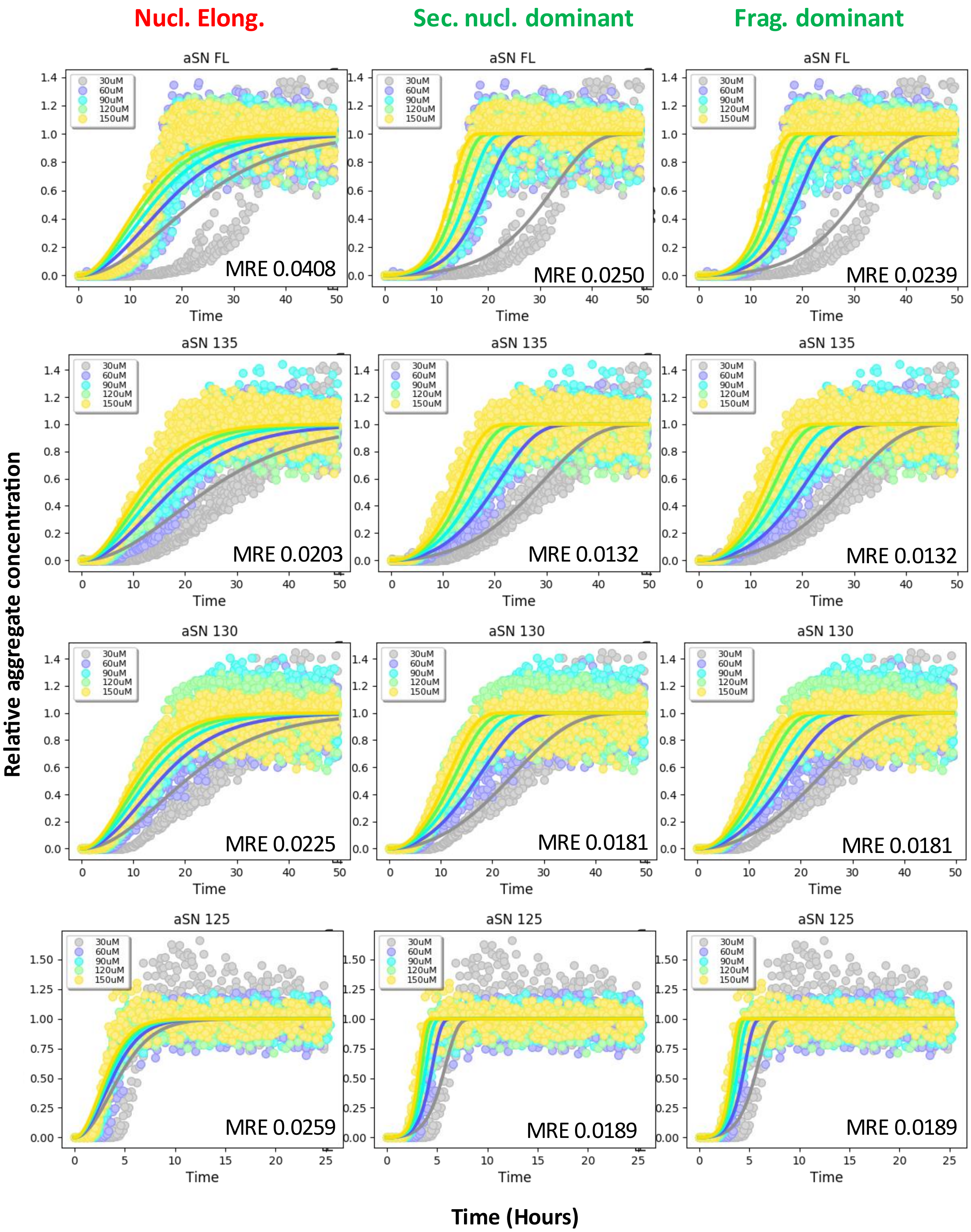

Figure S2. The best models to fit kinetic data for αSN-FL to αSN125 are the secondary nucleation dominant model and the fragmentation dominant model, while the nucleation and elongation model provides a poor fit. Note similar models with saturation (i.e., elongation saturation and secondary nucleation dominant, and elongation saturation and fragmentation dominant) fit just as well. This

indicates that saturation does not play a significant role within the measured concentration ranges. Three replicates in three wells were used at every concentration.

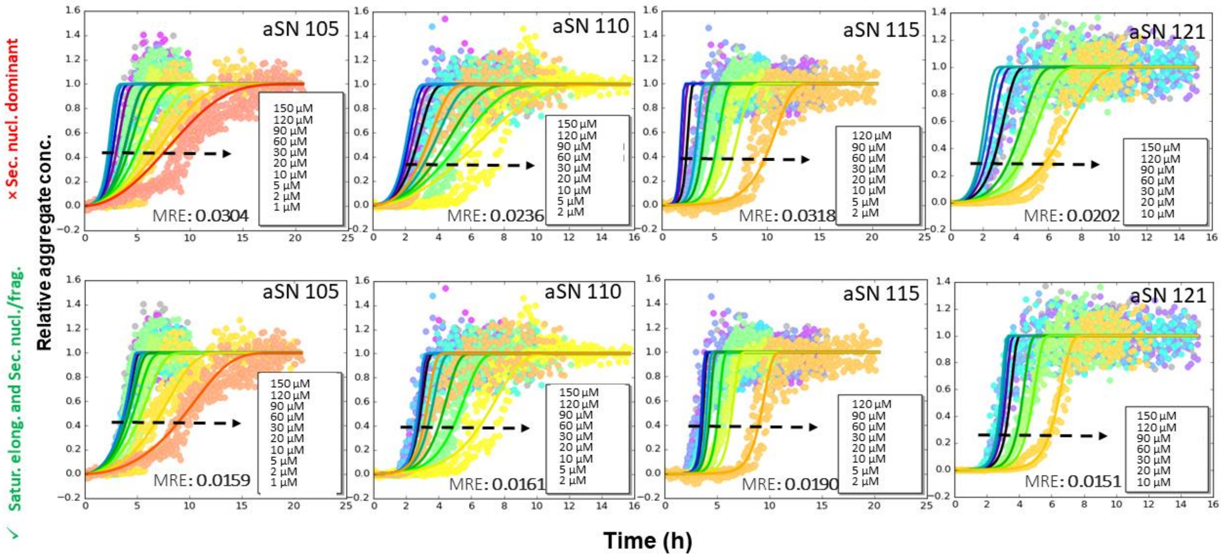

Figure S3. Comparison of "fragmentation" model (top) with "saturation elongation and fragmentation" (bottom) for αSN mutants shorter than αSN125. Visual inspection along with values of Mean Residual squared Error (MRE) provided for each fit show a better fitting when the saturation parameter is involved. Stippled arrow indicates direction of increasing protein concentration. Three replicates in three wells were used for every concentration.

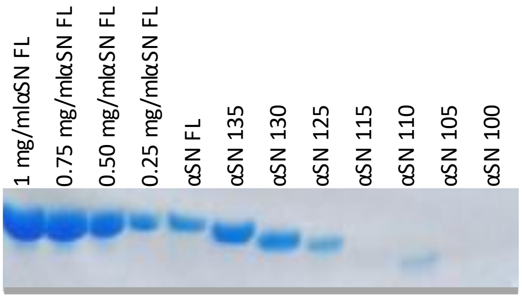

Monomer conc. (mg/ml)	0.24	0.34	0.3	0.15	0	0.07	0	0
Fibril conc. (mg/ml)	0.76	0.66	0.7	0.85	1	0.93	1	1

Figure S4. Determination of the extent of aggregation of different αSN mutants. The concentration of aggregates remaining at the end of the fibrillation experiments was measured by spinning the solution down and determining [monomer] by SDS-PAGE using a calibrated monomer sample. The fibril concentration was obtained by subtracting [monomer] from total [αSN]. The data show that there is overall a very high level of fibrillation and that truncation increases this even further.

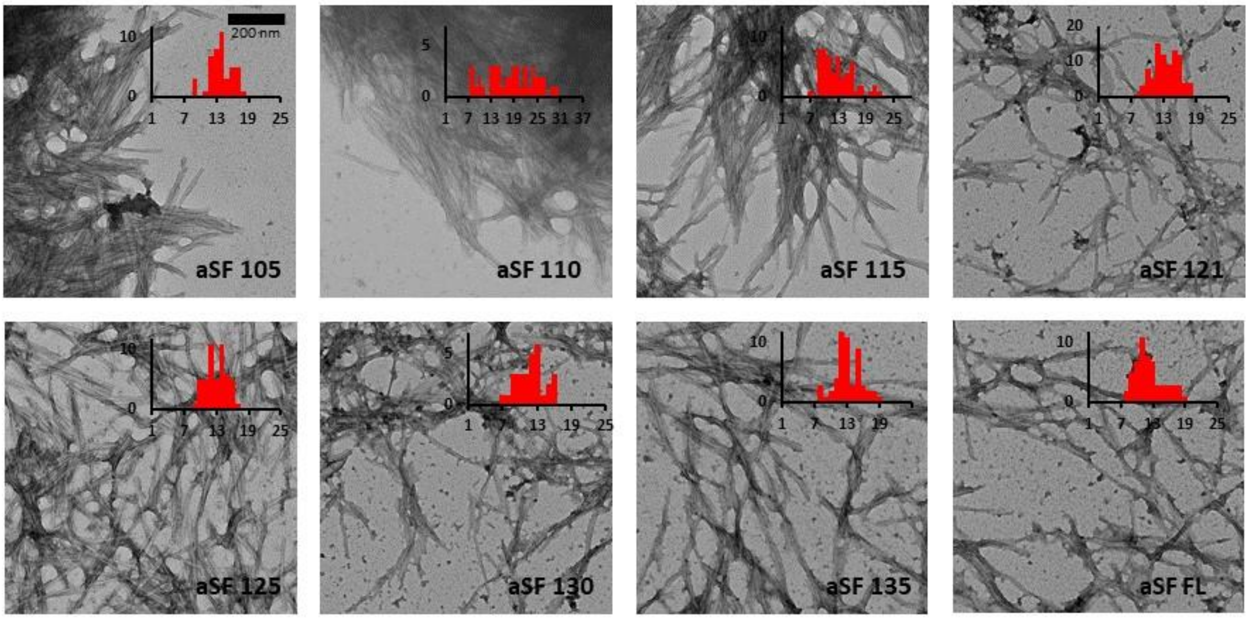

Figure S5. Negative stain TEM images of truncated αSN fibrils (αSF). Insets show frequency (y-axis) of fibrils width in nanometer (x-axis) quantified manually with ImageJ. Scale bar shows 200 nm.

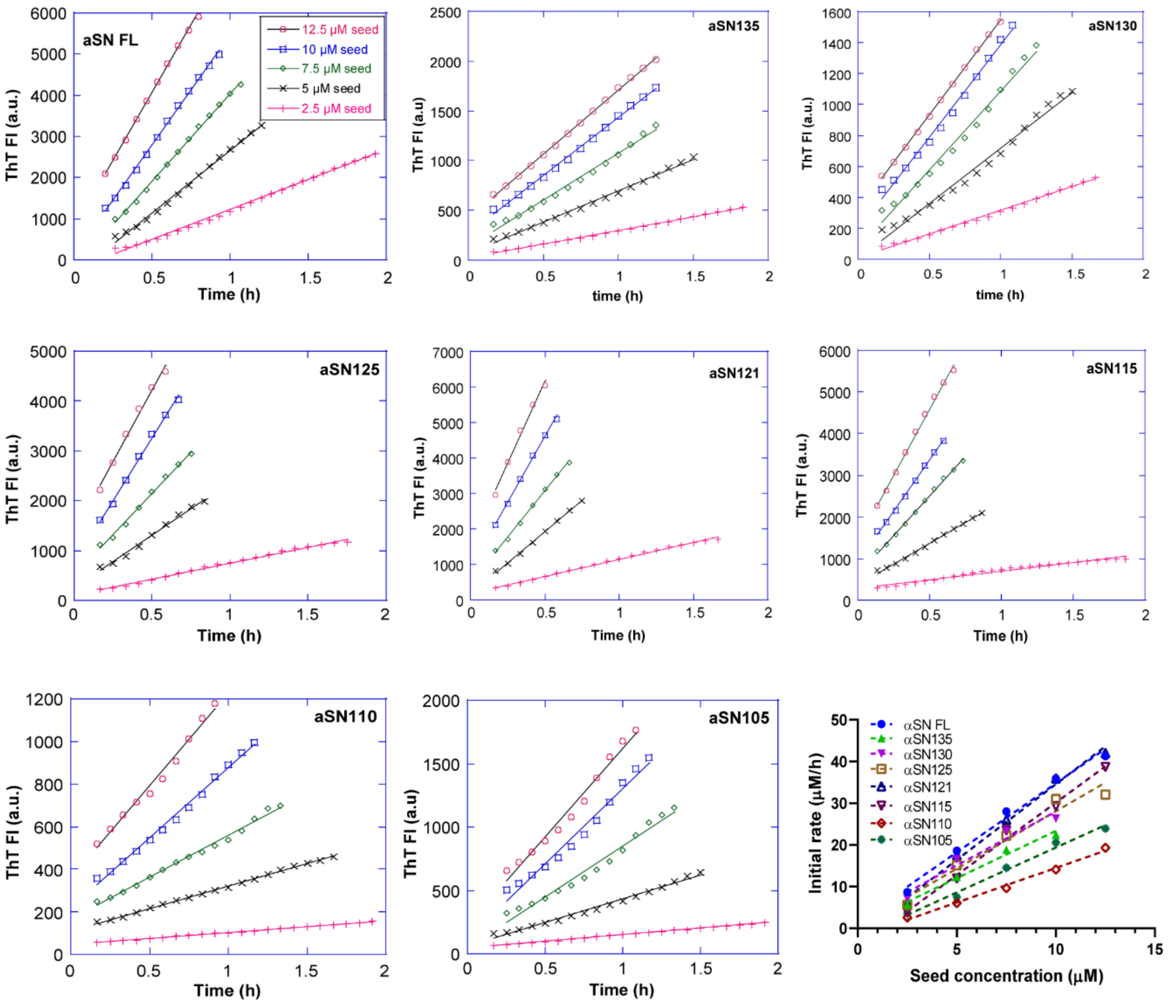

	Apparent elongation rate constant (slope)	Std. error	R^2	Relative elongation rate constant
**αSN-FL**	3.30	0.21	0.99	1
**αSN135**	2.26	0.20	0.98	0.7
**αSN130**	2.58	0.41	0.95	0.8
**αSN125**	2.75	0.34	0.96	0.9
**αSN121**	3.63	0.21	0.99	1.2
**αSN115**	3.48	0.17	0.99	1.4
**αSN110**	1.66	0.09	0.99	0.6
**αSN105**	2.15	0.14	0.99	0.9

Figure S6. Determination of elongation rates for each construct of αSN. A ThT fibrillation assay was performed in the presence of a series of seed concentrations (corresponding to 2.5 to 12.5 μM in monomer units) and a fixed concentration of monomer (50 μM). In the last panel, the initial rates of fibrillation were plotted against seed concentration to obtain the apparent elongation rate constant. Each curve is an average of two repeats. Earlier time points were excluded due to signal

changes originating from thermal equilibration. Results of these fits are summarized in the Table underneath the graphs. Relative elongation rate constant is obtained from apparent elongation rate constant where saturation coefficient (K_E) as Michaelis constant for elongation is taken into account (For more detail, see Material and Methods in the section "Analysis of fibrillation kinetics").

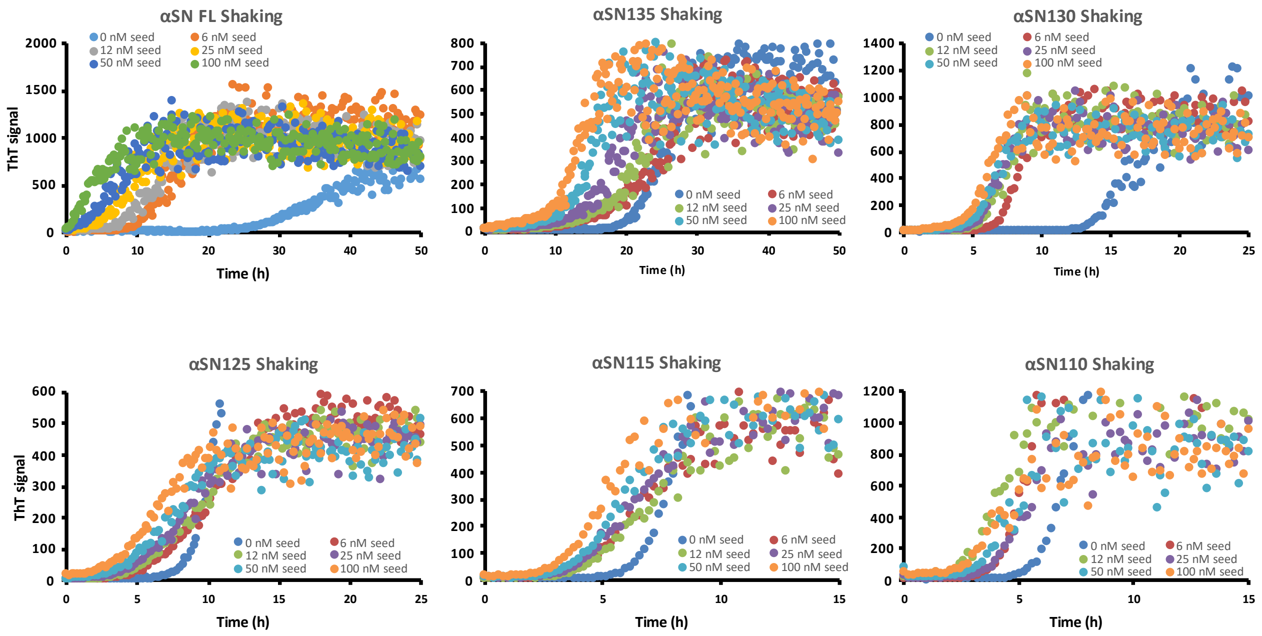

Figure S7. Fibrillation at low seed concentrations with shaking induces faster aggregation for aSN-FL as well as all CTD truncated mutants. Experiments were done with 50 µM monomer and the mentioned concentrations of seeds corresponding to 0, 0.03, 0.06, 0.1, and 0.2% seed in PBS pH 7.4.

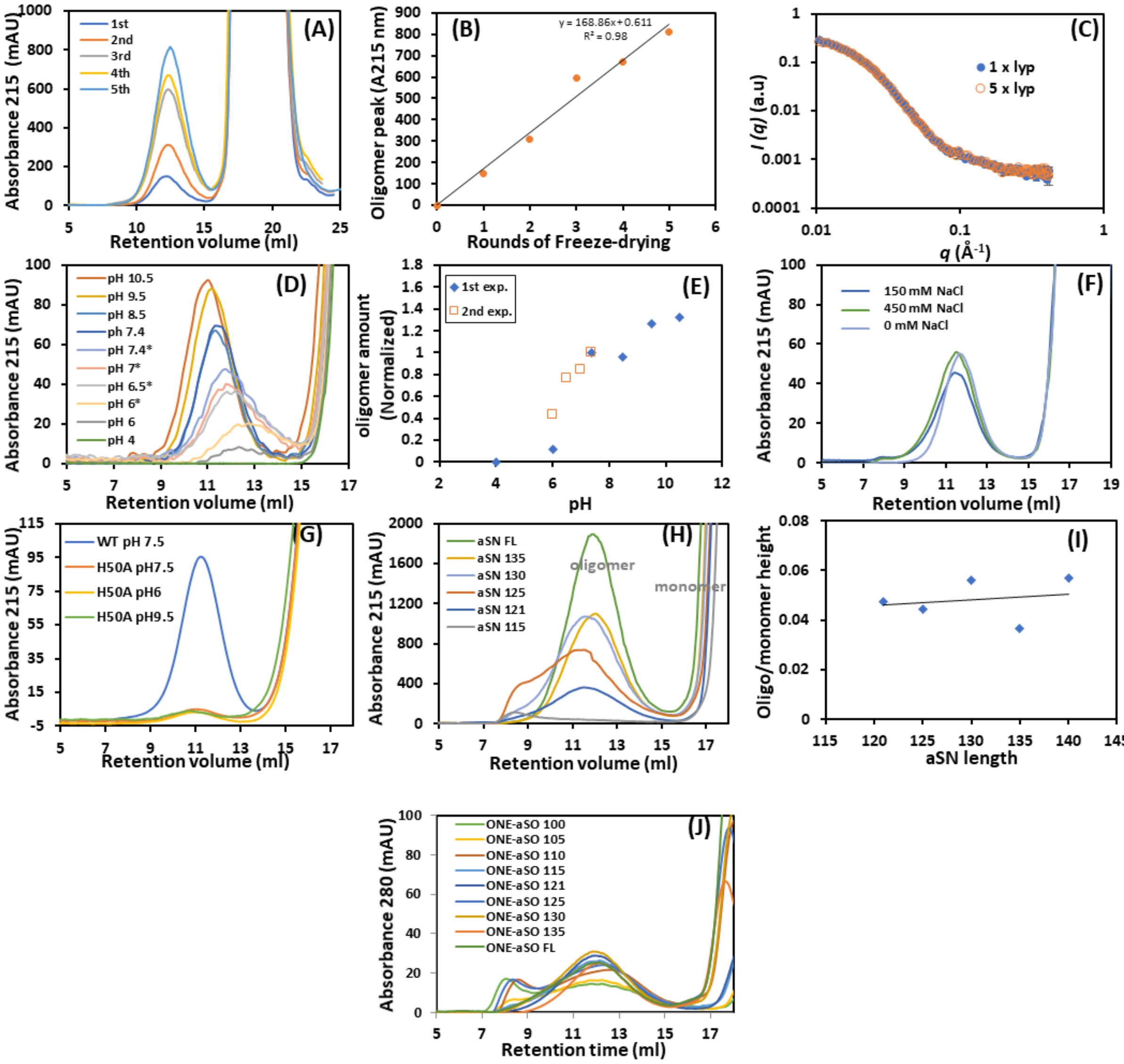

Figure S8. Oligomer formation tendency of C-terminal truncated αSN constructs based on size exclusion analysis. Data in panels A-F employ αSNFL. (A, B) Each round of freeze-drying led to a linear increase in the amount of αSN oligomer (αSO). (C) Oligomers of first and fifth rounds of freeze-drying show no difference in size and shape according to SAXS data. (D, E) Oligomer formation over a range of pHs from pH 10.5 to pH 4 shows almost no oligomer below pH 6. Asterisks in panel D refer to another set of experiments with a different batch of starting monomers (F) Oligomer formation in 20 mM phosphate buffer with three different NaCl concentrations. (G) H50A αSN does not form oligomers between pH 6 and 9.5 after 4 hours incubation in 37 C, 900 rpm shaking. (H) Shorter constructs do not form oligomer even after three rounds of freeze-drying. (I) The αSN constructs that form oligomer retain a constant proportion of oligomer to monomer based on peak height. (J) Modification with ONE induces oligomerization of all truncated αSNs, leading to oligomers that elute around 12 ml just like unmodified oligomers. We also observed

variable amounts of a larger species eluting around 8 ml (quantified as the ratio between the peak heights of oligomer and monomer fractions) but without any systematic trends in their formation.

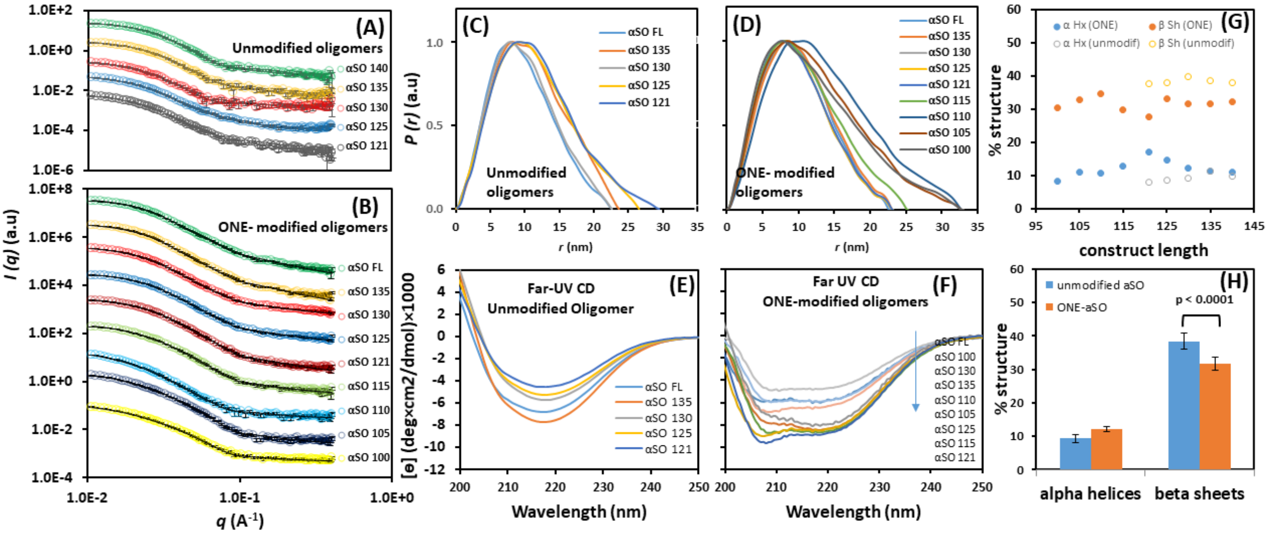

Figure S9. (A, B) Good fits (lines) to SAXS data of a previously developed model for the αSN-FL oligomer indicate a similar ellipsoidal shape for all oligomers with a dense core and fluffy shell. (C, D) Model-free pair distance distribution function ($p(r)$) shows generally similar ONE- and unmodified oligomers but more elongated ONE-oligomers for the three shortest αSN constructs. (E, F) secondary structures (CD curves) of unmodified and ONE-modified oligomers of CTD truncated αSN constructs and (G) deconvolution of the CD data into components of secondary structure (http://bestsel.elte.hu/). (H) β content of ONE-αSOs is slightly lower than for unmodified aSOs (D). One-way ANOVA was used to compare the secondary structure amounts in oligomer types in panel H. Error bars show the standard deviation for the average in each group.

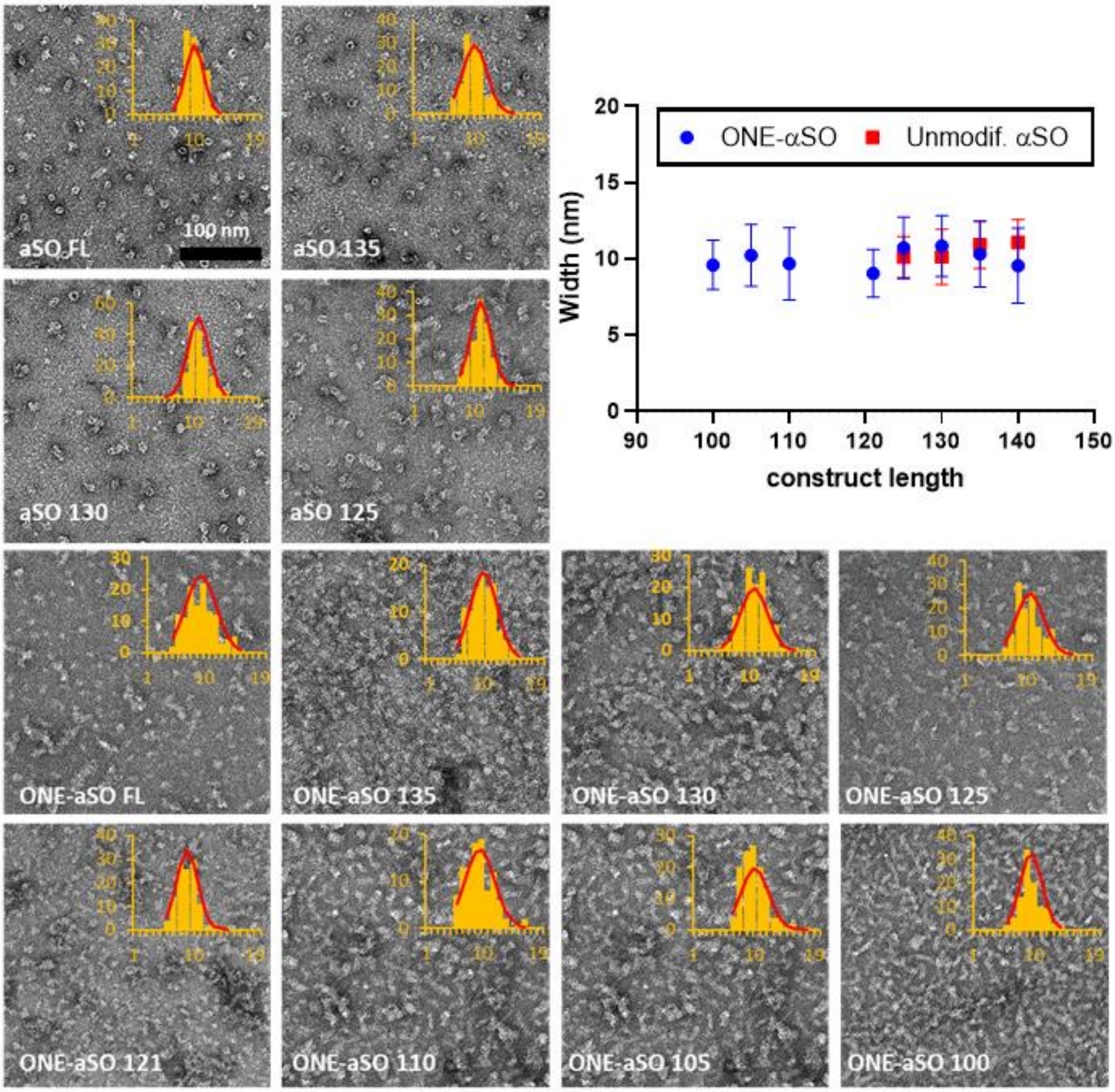

Figure S10. Negative stain TEM images of truncated αSOs. Insets show frequency (y-axis) of oligomer width in nanometer (x-axis) quantified manually with ImageJ. Scale bar shows 100 nm. The plot shows the width of different oligomers (data points are average of measured width and error bars are standard deviation).

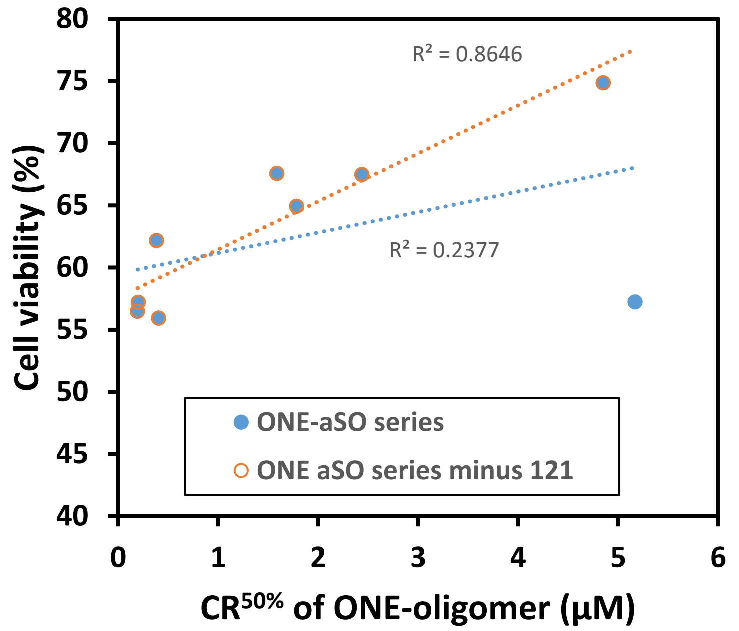

Figure S11. We observe a good correlation between $CR^{50\%}$ values of ONE-oligomers and cell viability with the single exclusion of ONE-αSO121.
