# Supplementary material for: The C-terminal tail of α-synuclein protects against aggregate replication but is critical for oligomerization
Source: Commun Biol. 2022 Feb 10;5:123. doi: 10.1038/s42003-022-03059-8 (PMC8831632; doi:10.1038/s42003-022-03059-8)
Supplement: Supplementary file 5 — Description of Additional Supplementary Files [file 42003_2022_3059_MOESM5_ESM.pdf]

# Description of Additional Supplementary Files

**File name:** Supplementary Data 1
**Description:** Data sets for graphs and charts in the main figures.
